# Treatment Outcome of External Auditory Canal Carcinoma: The Utility of Lateral Temporal Bone Resection

**DOI:** 10.3389/fsurg.2021.708245

**Published:** 2021-08-30

**Authors:** Kohei Saijo, Yushi Ueki, Ryoko Tanaka, Yusuke Yokoyama, Jo Omata, Takeshi Takahashi, Hisayuki Ota, Ryusuke Shodo, Keisuke Yamazaki, Takafumi Togashi, Ryuichi Okabe, Hiroshi Matsuyama, Kohei Honda, Yuichiro Sato, Yuka Morita, Kuniyuki Takahashi, Arata Horii

**Affiliations:** ^1^Department of Otolaryngology Head and Neck Surgery, Niigata University Graduate School of Medical and Dental Sciences, Niigata, Japan; ^2^Department of Head and Neck Surgery, Niigata Cancer Center Hospital, Niigata, Japan; ^3^Department of Otolaryngology, Nagaoka Red Cross Hospital, Nagaoka, Japan; ^4^Department of Otolaryngology, Nagaoka Chuo General Hospital, Nagaoka, Japan; ^5^Department of Otolaryngology, Niigata General Hospital, Niigata, Japan; ^6^Department of Otolaryngology, Niigata Prefectural Central Hospital, Joetsu, Japan

**Keywords:** lateral temporal bone resection, external ear canal carcinoma, negative surgical margin, preoperative therapy, postoperative therapy

## Abstract

We examined the role of lateral temporal bone resection (LTBR) in the treatment of external ear canal (EAC) carcinoma between 2007 and 2018. The estimated 3-year disease-free survival (DFS) and disease-specific survival (DSS) according to the tumor stage and treatments were investigated in 36 patients with EAC squamous cell carcinoma. T stage classification according to the University of Pittsburgh staging system was as follows: 14 patients in T1, four patients in T2, nine patients in T3, and nine patients in T4. The 3-year DFS rate was 77.4% for T1 tumors, 100% for T2, 44.4% for T3 tumors, and 11.1% for T4 tumors (*p* < 001). The 3-year DSS rate was 100% for T1/T2 tumors, 87.5% for T3 tumors, and 11.1% for T4 tumors (*p* < 0.01). T1/T2 patients received mostly LTBR. Among nine T3 tumors, five patients (56%) received LTBR combined with preoperative chemotherapy and/or postoperative radiation (RT). Four of them had negative surgical margin and survived with no evidence of disease. The DFS of T3 patients who underwent concurrent chemoradiotherapy and LTBR was 0 and 80%, respectively (*p* = 0.048). For T1/T2 tumors, surgery achieved an excellent outcome. For T3 tumors, LTBR achieved negative surgical margin and showed good survival when combined with preoperative chemotherapy and/or postoperative RT. In contrast, the prognosis of T3 patients who could not undergo surgery was as poor as that of T4 patients. Therefore, in addition to subtotal temporal bone resection, LTBR-based treatment strategy may be a treatment option for limited cases of T3 patients.

## Introduction

Temporal bone carcinoma is a rare disease that accounts for <0.2% of all head and neck cancers (1). Most temporal bone carcinomas originate from the external auditory canal (EAC), and the most common histological type is squamous cell carcinoma (2). The University of Pittsburgh staging system has been widely used for the classification of tumor extension in EAC carcinoma (3), which is well-associated with treatment outcomes (4). Although there have been no large cohort studies due to its rarity, complete surgical resection is considered to be an optimal treatment (5). For this purpose, sleeve resection or lateral temporal bone resection (LTBR) is suitable for T1/T2 tumors that are localized to the EAC and EAC with bone erosion or with <0.5 mm soft tissue involvement, for which favorable outcomes have been reported (6, 7). On the other hand, subtotal temporal bone resection (STBR) has been proposed as a standard surgery for advanced carcinoma, which is a more invasive and challenging approach than LTBR because of its anatomical complexity (5, 8). As a result, STBR is being performed in relatively few hospitals and is replaced by concurrent chemoradiotherapy (CCRT) in the treatment of advanced tumors.

As an alternative to STBR, LTBR may be adopted in limited T3/T4 cases in combination with preoperative chemotherapy and/or radiotherapy (RT)/CCRT in clinical settings (8–11). Because LTBR is safe, less invasive, and able to be performed in most hospitals, the role of LTBR in treating advanced EAC carcinoma should be revisited. In this retrospective study, we aimed to evaluate the treatment outcomes of EAC carcinoma and the role of LTBR in the treatment of EAC carcinoma.

## Materials and Methods

### Patients

This study was approved by the Institutional Review Board of Niigata University Hospital (No. 2019–0171). All patients provided written informed consent for participation in the study. This study was conducted in accordance with the principles of the Declaration of Helsinki. Between 2007 and 2018, 52 patients with auricle, external auditory canal, and middle ear carcinoma were treated at our institute. Among them, we retrospectively analyzed 36 patients with squamous cell carcinoma arising from the EAC. We reviewed the electronic clinical records and extracted data for age, sex, staging according to the University of Pittsburgh system (3), treatment history, histological findings, and treatment outcomes. Moreover, we estimated disease-free survival (DFS) and disease-specific survival (DSS) as treatment outcomes. DFS was defined as the time from the date of starting the initial treatment to the date of disease progression or death, and DSS was defined as the time from the date of starting the initial treatment to the date of death related to the disease.

### Treatment Strategy

**Figure 1:**
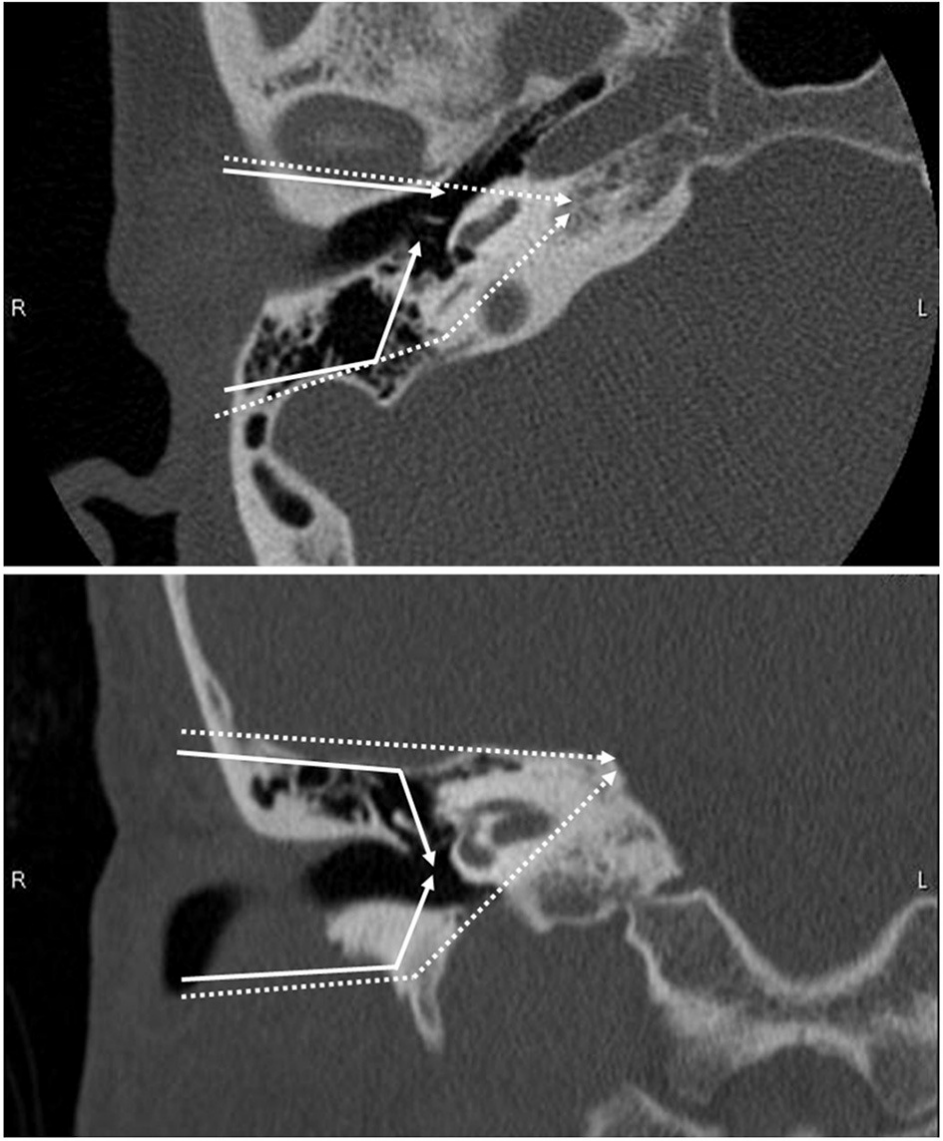
Schematic CT images showing the resection line of lateral temporal bone resection. **Upper panel**: axial image. **Lower panel**: coronal image. Solid line shows the resection line of LTBR. Dotted line shows the resection line of STBR.

T1 and T2 tumors were mostly treated with surgery. Sleeve resection, which removes only the skin of the EAC, was selected for tumors limited to EAC without bony erosion, while LTBR was used for the others. The LTBR procedure includes en bloc resection of the EAC with the tympanic membrane and complete mastoidectomy. STBR needs the resection of otic capsule in addition to LTBR. Schematic CT images of the resection area in the standard LTBR and STBR are shown in Figure 1. According to the tumor extension, additional resections of soft tissue such as parotidectomy and resection of the temporomandibular joint and facial nerve were combined. The surgical defect was reconstructed using the temporal fascia, temporal muscle flap, or anterolateral thigh musculocutaneous flap without ossicular chain reconstruction. Elective neck dissection was not performed routinely, except in cases that require a free flap reconstruction. If postoperative pathological examination revealed a positive surgical margin, postoperative RT was performed.

Definitive CCRT was performed in patients with T3/T4 tumors that invaded the middle ear cavity, tympanic membrane, petrous apex, carotid canal, or dura and patients with difficulties in undergoing LTBR due to anatomical complexity (e.g., sclerotic mastoid or post-mastoidectomy). LTBR was chosen for the other T3/T4 cases, with or without preoperative chemotherapy and postoperative RT/CCRT. Preoperative chemotherapy included cisplatin (60 mg/m^2^, day 1) plus 5-fluorouracil (600 mg/m^2^, day 1–5). RT was performed using 3-dimensional conformal RT and stereotactic radiotherapy (SRT). The total dose was 70 Gy/35 fraction in definitive RT or CCRT and 60 Gy/30 fraction in postoperative RT. The biological effective dose of SRT was 42 Gy or more, and the dose per fraction was 4 Gy or more. The regimen of chemotherapy in CCRT consisted of three cycles of cisplatin (80 mg/m^2^) or two cycles of cisplatin (60 mg/m^2^, day 1) plus 5-fluorouracil (600 mg/m^2^, day 1–5). Those who were ineligible for CCRT due to poor general condition received SRT.

### Statistical Analysis

The cut-off date for the analyses of DFS and DSS was December 31st, 2019. The median follow-up time of the patients was 34 months (range, 4–142 months). Survival time was estimated using the Kaplan-Meier method and compared using log-rank tests. All statistical analyses were performed using EZR (Saitama Medical Center, Jichi Medical University, Saitama, Japan), which is a graphical user interface for R (The R Foundation for Statistical Computing, Vienna, Austria). More precisely, it is a modified version of R commander designed to add statistical functions frequently used in biostatistics (12).

## Results

The median age of the 36 patients was 63 years (36–86 years). Twenty-two patients (61.1%) were men, and 14 patients (38.9%) were women. T stage classification according to the University of Pittsburgh staging system was as follows: 14 patients in T1, four patients in T2, nine patients in T3, and nine patients in T4. No patient had lymph node metastasis or distant metastasis at the initial diagnosis.

Table 1 shows the details of T1/T2 patients. All 14 T1 patients were treated by surgery: four and 10 patients underwent sleeve resection and LTBR, respectively. Among the four T2 patients, three underwent LTBR and the other received RT alone because of poor general condition. All four patients with positive or close surgical margins received postoperative RT or CCRT and survived without recurrence. Two T1 patients had recurrences: one had a local recurrence who was rescued by salvage CCRT. The other had a nodal recurrence who was successfully treated with neck dissection. At the cutoff date, all T1/T2 patients survived without disease or died of other causes.

**Table 1:** Treatment details in T1 and T2 patients.

**T stage**	**Sex**	**Age**	**Treatment**	**Margin**	**RT dose**	**Recurrence**	**Follow-up** **(months)**	**Status**
1	M	66	Sleeve resection → RT	Positive	60	None	140	NED
	M	57	Sleeve resection → CCRT	Close margin	60	None	94	NED
	F	67	Sleeve resection	Negative		None	52	NED
	M	74	Sleeve resection	Negative		None	28	DOO
	F	83	LTBR	Negative		N	59	NED
	M	82	LTBR → RT	Positive	60	None	89	NED
	M	55	LTBR	Negative		None	61	NED
	F	62	LTBR	Negative		None	93	NED
	F	36	LTBR	Negative		None	69	NED
	M	65	LTBR	Negative		None	70	NED
	M	83	LTBR	Negative		None	46	NED
	F	62	LTBR	Negative		None	42	NED
	F	72	LTBR	Negative		T	38	NED
	M	62	LTBR	Negative		None	24	NED
2	F	76	LTBR	Indeter-minable		None	61	NED
	M	79	LTBR → RT	Positive	66	None	28	NED
	F	51	LTBR → RT	Indeter-minable	60	None	21	NED
	F	80	RT	Negative	50	None	48	DOO

*LTBR, lateral temporal bone resection; RT, radiotherapy; CCRT, concurrent chemoradiotherapy; NED, no evidence of disease; DOD, died of disease; DOO, died of other cause*.

Table 2 shows the details of T3/T4 patients. Among the nine T3 patients, five and four patients underwent LTBR and CCRT, respectively. Among the five patients who received LTBR, three patients received neo-adjuvant chemotherapy, and four patients underwent postoperative RT/CCRT. The reasons that the other four T3 patients received CCRT were as follows: one had tumor extension to anterior wall of EAC close to carotid canal, one had middle ear cavity invasion, and the other two had difficulties in performing mastoidectomy due to sclerotic mastoid or post-mastoidectomy state. Among the five T3 patients who underwent LTBR, three patients received additional resections of adjacent tissues (parotid gland/temporomandibular joint/facial nerve). Except for one patient who had a positive surgical margin in the middle ear cavity, four of five T3 patients who underwent LTBR achieved negative surgical margin and survived without recurrence or metastasis. In contrast, all four T3 patients who received definitive CCRT experienced recurrence or metastasis. Among the nine T4 patients, one, two, and six patients underwent LTBR followed by postoperative RT, SRT alone, and definitive CCRT, respectively. One T4 patient who underwent LTBR followed by RT died due to early local recurrence. Among eight patients who received CCRT or SRT, seven had locoregional recurrence or metastasis. The other patient survived without recurrence or metastasis. The T4 tumor of this patient extended to the lateral subcutaneous tissue, including the auricle, without invasion of the medial side of the temporal bone.

**Table 2:** Treatment details in T3 and T4 patients.

**T stage**	**Sex**	**Age**	**Site of extension**	**Treatment**	**Resection**	**Margin**	**RT** ** dose**	**Recurrence**	**Follow-up** ** (months)**	**Status**
3	F	60	Mandibular joint	NAC → LTBR → RT	Mandibular joint	Negative	60	None	142	NED
	F	48	Mastoid, parotid gland	NAC → LTBR → RT	Parotid gland, mandibular joint, facial nerve	Negative	60	None	73	NED
	M	63	Mastoid	LTBR → RT	None	Negative	60	None	37	NED
	M	56	Mastoid	NAC → LTBR	Parotid gland, facial nerve	Negative	none	None	34	NED
	M	56	Mastoid	LTBR → CCRT(CDDP)	None	Middle ear	60	T,N	10	AWD
	M	75	Anterior wall of external auditory tract	CCRT (CDDP+5-FU)			70	N	84	NED
	F	49	Mastoid, middle ear	CCRT (CDDP+5-FU)			70	M	18	DOD
	M	69	Mastoid	CCRT (CDDP)			70	T	29	AWD
	M	63	Mastoid	CCRT (CDDP)			70	T	25	AWD
4	M	75	Styloid process	LTBR → RT	Parotid gland	Around styloid process	66	T	4	DOD
	M	53	Medial wall of middle ear	SRT			63	T,M	10	DOD
	M	86	Mandibular joint	SRT			40	NA	6	DOO
	M	58	Dura	CCRT (CDDP+5-FU)			70	N	13	DOD
	M	59	Soft tissue (extensive subcutaneous tissue involvement)	CCRT (CDDP)			70	None	21	NED
	M	74	Dura, mandibular joint	CCRT (CDDP)			70	T	6	DOD
	F	55	Dura	NAC → CCRT(CDDP)			66	T,N,M	10	DOD
	F	58	Petrous apex, dura, mandibular joint	CCRT (CDDP+5-FU)			70	T	7	DOD
	M	53	Parotid gland	CCRT (CDDP)			70	T	12	DOD

*NAC, neoadjuvant chemotherapy; LTBR, lateral temporal bone resection; RT, radiotherapy; CCRT, concurrent chemoradiotherapy; SRT, stereotactic radiotherapy; NED, no evidence of disease; AWD, alive with disease; DOD, died of disease; DOO, died of other cause; NA, not available*.

**Figure 2:**
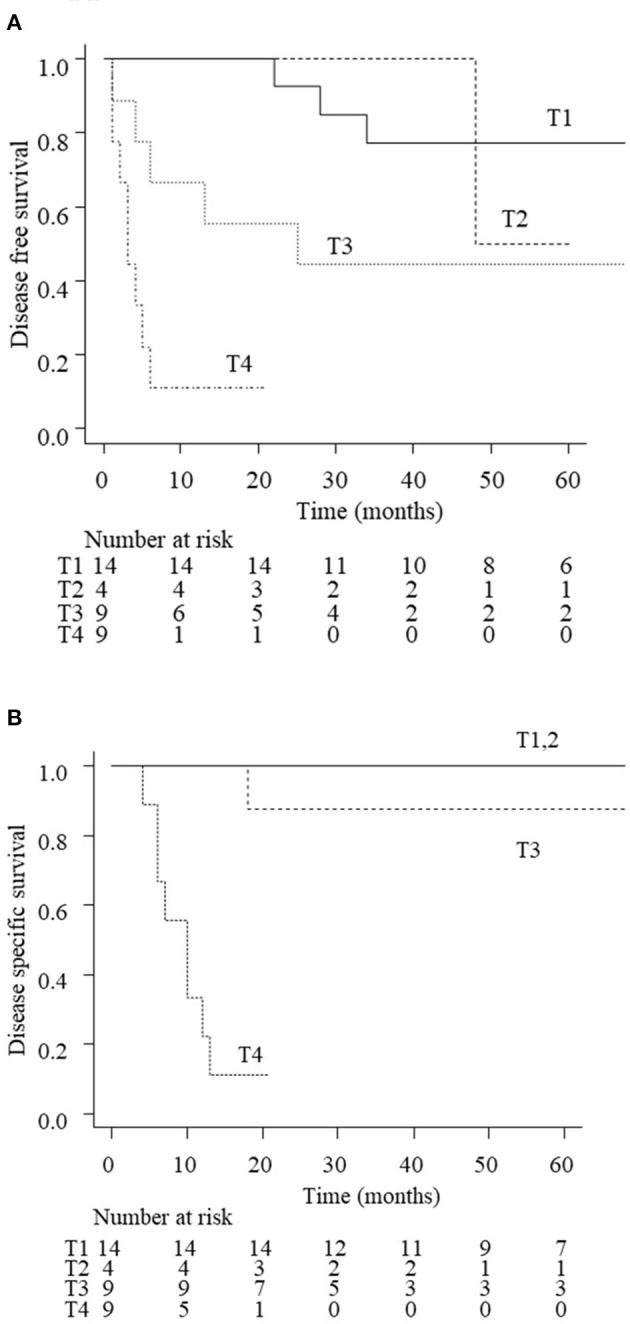
Disease free survival (DFS; **A**) and disease specific survival (DSS; **B**) of EAC carcinoma patients according to the disease stage. The estimated 3-year DFS rate of all patients was 77.4% in stage 1, 100% in stage 2, 44.4% in stage 3, and 11.1% in stage 4 (*p* < 0.01). The estimated 3-year DSS rate of all patients was 100% in stages 1 and 2, 87.5% in stage 3, and 11.1% in stage 4 (*p* < 0.01).

**Figure 3:**
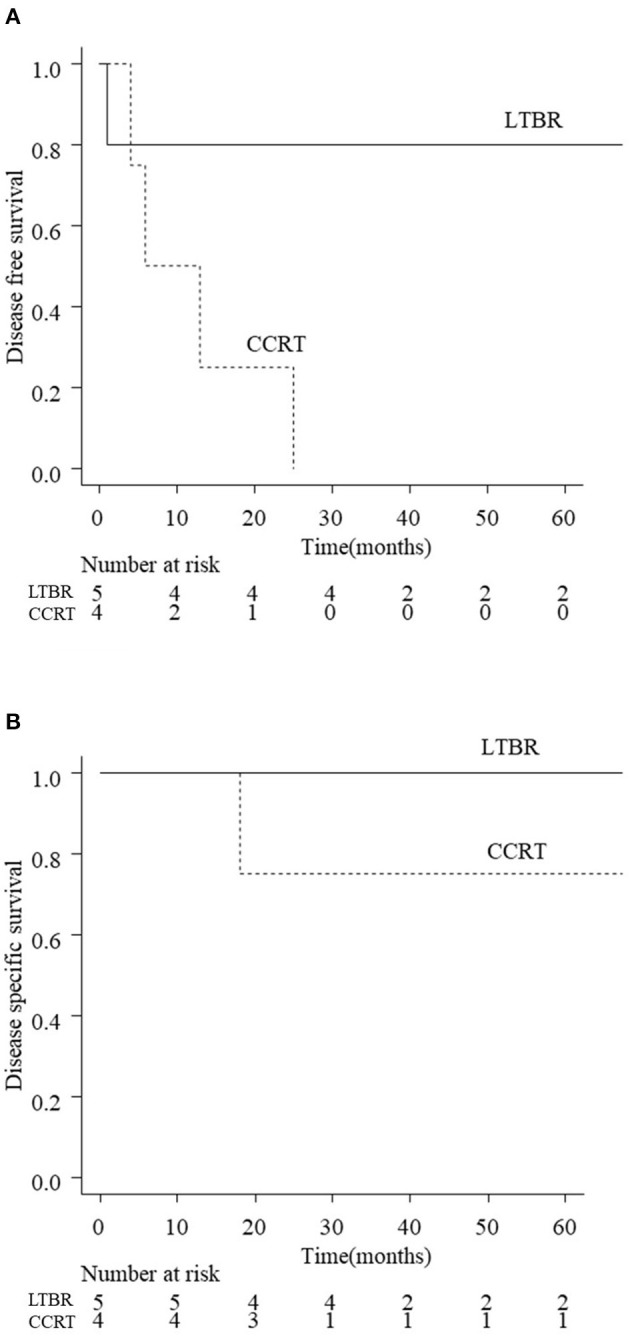
Disease free survival (DFS; **A**) and disease specific survival (DSS; **B**) of T3 patients according to the treatments (LTBR vs. CCRT). The estimated 3-year DFS rates of T3 patients in the LTBR group and CCRT group were 80 and 0%, respectively (*p* < 0.05). The estimated 3-year DSS rates of T3 patients in the LTBR group and CCRT group were 100 and 75%, respectively (*p* = 0.317). LTBR, lateral temporal bone resection; CCRT, concurrent chemoradiotherapy.

The 3-year DFS and DSS rates for all patients with EAC carcinoma were 56.1 and 74.6%, respectively. Figure 2 shows the treatment outcomes according to the tumor stage. The 3-year rate of DFS was 77.4% with T1 tumors, 100% with T2, 44.4% with T3, and 11.1% for T4 tumors, respectively (*p* < 001). The 3-year rate of DSS was 100% for T1/T2 tumors, 87.5% for T3, and 11.1% for T4 (*p* < 0.01). The survival curves of T3 patients according to the treatment (LTBR or CCRT) are shown in Figure 3. The 3-year rate of DFS with T3 tumors was 80% in the LTBR group and 0% in the CCRT group (*p* < 0.05). The 3-year rate of DSS rate was 100% in the LTBR group and 75% in the CCRT group (*p* = 0.317).

## Discussion

The present study shows that the staging system of the University of Pittsburgh is useful for predicting the prognosis of EAC carcinoma (Figure 2). For T1/T2 tumors, the 3-year rate of DSS was 100%, demonstrating a favorable outcome of en bloc resection using sleeve resection or LTBR. These results are consistent with those of previous studies demonstrating good prognosis of early stage EAC carcinoma (7, 11, 13, 14). According to the NCCN guideline of head and neck cancers, positive margin cases for T1 patients should receive postoperative CCRT (15). However, EAC carcinoma is not included in the guideline due to its rarity. Bacciu et al. recommended RT for T1/T2 EAC carcinoma patients with positive surgical margins (11). We chose postoperative RT rather than CCRT to achieve less invasive treatment based on this report. The present results, which demonstrated that all four patients who had positive or close surgical margins could be rescued by postoperative RT, were consistent with this study. It is suggested that sleeve resection or LTBR is recommended for T1/T2 tumors and that postoperative RT should be added in cases with positive surgical margins.

For T3/T4 tumors, STBR is the standard procedure that enables en bloc resection of the tumor. However, STBR is a much more challenging approach than LTBR because of its anatomical complexity (5, 8) and is sometimes replaced by CCRT. LTBR combined with preoperative chemotherapy/postoperative RT may be adopted for limited cases of T3/T4 tumors. Therefore, we compared the treatment outcome between CCRT and LTBR-based treatment for T3/T4 tumors. The present results showed that the treatment outcome of T3 EAC carcinoma differed greatly according to the treatment strategy (CCRT or LTBR) (Figure 3A). In the present study, five of nine T3 patients received LTBR with preoperative chemotherapy and/or postoperative RT. Four out of five T3 patients who received LTBR (80%) survived without recurrence. The 3-year DFS rate after CCRT for T3 tumors (0%) was significantly lower than that of LTBR (80%) (Figure 3A). One T4 patient who received LTBR died due to early local recurrence. These results suggest that LTBR but not STBR can be considered for limited cases with T3 patients, while it would not be suitable for T4 patients.

Given that the two patients (one patient had T3 tumor with invasion of the middle ear cavity and another had T4 tumor) with positive surgical margins had early local recurrence (Table 2), negative surgical margins would be important for obtaining a successful result by LTBR in T3/T4 tumors. Previous reports have also shown that a positive surgical margin is a poor prognostic factor in the treatment of EAC carcinoma (11, 16, 17). Since LTBR usually cannot achieve an en bloc dissection of tumors in T3/T4 cases, preoperative therapy, additional surgeries such as parotidectomy, and resection of the skin/temporomandibular joint may be important in the management of T3 tumors using LTBR-based strategies. Regarding the additional treatment for LTBR for T3/T4 tumors, Komune et al. reported that LTBR with preoperative therapy (chemotherapy or CCRT) achieved negative surgical margins in 10 of 14 T3 patients and seven of ten T4 patients, respectively (8). In a meta-analysis, Takenaka et al. reported that preoperative CCRT was significantly associated with good prognosis in patients with advanced EAC carcinoma (18). In general, preoperative chemotherapy and preoperative CCRT are not standard treatments for head and neck cancer. Because LTBR is a typical procedure including mastoidectomy, clearer surgical margins can be stably obtained. Moreover, LTBR can be performed in combination with various surgeries, including parotidectomy and resection of the skin or temporomandibular joint. Our results and these reports suggest that LTBR combined with preoperative therapy and/or additional resection of adjacent tissues could be applied to limited cases of T3 EAC carcinoma that does not invade deep regions such as the middle cranial fossa, eustachian tube, carotid canal, and petrous apex. As a second line treatment for those who are unfit or contra-indicated for STBR, LTBR-based strategy may be a treatment option for limited patients with T3 tumors. In contrast to T1/T2 tumors with positive surgical margins, postoperative RT was not effective for T3/T4 patients who had positive surgical margins by LTBR (Table 2). Judging the possibilities of complete resection was quite important when performing LTBR in T3 patients.

Both local control and survival rates of T3/T4 patients who underwent CCRT were very poor (Table 2, Figure 3A). In principle, STBR should be performed in patients with T3 tumors who are unfit to undergo LTBR (complete resection cannot be performed by mastoidectomy or the tumor invades deeper than the middle ear) or in T4 tumors. However, as mentioned above, STBR is more invasive than LTBR. Therefore, CCRT must be performed for patients who are not fit for surgery due to poor general condition or in whom the tumor cannot be resected even with STBR. In this study, a cisplatin-based regimen was mainly used for CCRT, according to the standard therapy for head and neck carcinoma. As an alternative to cisplatin-based regimens, the TPF regimen (docetaxel, cisplatin, and fluorouracil), which enhances the intensity of CCRT (19–21), may be considered for the management of unresectable T3/T4 tumors. TPF as an induction chemotherapy was shown to achieve significantly better survival than cisplatin and fluorouracil in unresectable head and neck cancer (22, 23). Shiga et al. reported that the overall 5-year survival rates of patients with T3 or T4 EAC carcinoma who underwent CCRT with TPF and cisplatin regimens were 64.4 and 36.7%, respectively (21). These reports demonstrate that CCRT with TPF is a promising therapy for advanced EAC carcinoma, which is unfit for STBR.

As limitations of the study, this is a retrospective study that included non-uniform regimens for CCRT: The regimen of chemotherapy in CCRT consisted of three cycles of cisplatin (80 mg/m^2^) or two cycles of cisplatin (60 mg/m^2^, day 1) plus 5-fluorouracil (600 mg/m^2^, day 1–5). The latter regimen, which was used during an early period of the study, may be insufficient in comparison with the former regimen.

## Conclusion

Surgery treatment outcomes (sleeve dissection or LTBR) for T1/T2 EAC carcinoma were favorable. For limited cases of T3 cases, LTBR achieved negative surgical margin and showed good survivals when combined with preoperative chemotherapy and/or postoperative RT/CCRT. In contrast, the prognosis of T3 patients who could not undergo surgery and T4 patients was very poor. Therefore, in addition to STBR, LTBR-based treatment strategy may be a treatment option for limited cases of T3 tumors.

## Data Availability Statement

The original contributions presented in the study are included in the article/supplementary material, further inquiries can be directed to the corresponding authors.

## Ethics Statement

This study was approved by the Institutional Review Board of Niigata University Hospital (No. 2019–0171). All patients provided written informed consent for participation in the study. This study was conducted in accordance with the principles of the Declaration of Helsinki.

## Author Contributions

KS, YU, and AH: conception and design and drafting of the manuscript. KS, YU, RT, YY, JO, TTa, HO, RS, KY, TTo, RO, HM, KH, YS, YM, and KT: analysis and interpretation of data. YU: had full access to all the data in the study and takes responsibility for the integrity of the data and the accuracy of the data analysis. All authors final approval of the version to be published.

## Conflict of Interest

The authors declare that the research was conducted in the absence of any commercial or financial relationships that could be construed as a potential conflict of interest.

## Publisher's Note

All claims expressed in this article are solely those of the authors and do not necessarily represent those of their affiliated organizations, or those of the publisher, the editors and the reviewers. Any product that may be evaluated in this article, or claim that may be made by its manufacturer, is not guaranteed or endorsed by the publisher.
